# From mystery to modulation: the structural story of Rb_2_[Si_2_O_5_]

**DOI:** 10.1107/S205252062600819X

**Published:** 2026-09-01

**Authors:** Clivia Hejny, Volker Kahlenberg, Hannes Krüger

**Affiliations:** a Mineralogy and Petrography University of Innsbruck Innrain 52 Innsbruck 6020 Austria; Academy of Sciences of the Czech Republic, Czechia

**Keywords:** crenel function, incommensurately modulated crystal structure, Rb_2_Si_2_O_5_, phyllosilicate

## Abstract

The crystal structure of Rb_2_[Si_2_O_5_], a phyllosilicate with previously questionable structural details, has been solved and refined as an incommensurately modulated phase in (3 + 1)-dimensional superspace. This paper describes the monoclinic structure in superspace group *C*2/*c*(0β0)*s*0 with unit-cell parameters *a* = 9.8662 (6), *b* = 8.3986 (5), *c* = 14.7641 (9) Å, β = 90.114 (5)°, *V* = 1223.4 (1) Å^3^ and with modulation wavevector **q** = 0.377 (1)b*, refined to an *R*_1_ value of 0.0419 for 2603 reflections with intensities greater 3σ. All [SiO_4_] tetrahedra within the structure adopt two distinct orientations, and the positions of their constituent silicon and oxygen atoms are modeled using a combination of crenel and positional modulation functions. For the rubidium atoms, harmonic modulation waves were used for the atomic coordinates and the anisotropic displacement parameters. Rubidium cations are predominantly coordinated by six oxygen atoms, forming linkages between adjacent layers built of [SiO_4_] tetrahedra. However, the ionic radius of Rb in comparison to the mesh-size of the silicate layer leads to deformation and modulation of the layers. The modulation results in more balanced bond valence sums for rubidium, acceptable Si–O distances and anisotropic displacement parameters, which is not the case for a three-dimensionally periodic structure model with split atom positions. A comparison with related phyllosilicates featuring the same 4.8^2^ net layer topology, but with larger interlayer cations or higher cationic content, reveals that these structures exhibit more relaxed silicate layers. This highlights the role of cation size and content in influencing the structural modulation and strain within the crystal structure of phyllosilicates.

## Abstract 2

The crystal structure of Rb_2_[Si_2_O_5_] has been solved and refined as an incommensurately modulated phase. Each [SiO_4_] tetrahedron of the phyllosilicate adopts one of two distinct orientations, and the positions of their constituent silicon and oxygen atoms are modeled using a combination of crenel and harmonic positional modulation functions.

## 1. Introduction

Alkali silicates – primarily sodium and potassium silicates – play a significant role in various fields of inorganic chemistry and applied mineralogy (Weldes & Lange, 1969 ▸; Lagaly *et al.*, 2000 ▸; Zellmann & Kaps, 2006 ▸; Alavi *et al.*, 2021 ▸; Matinfar & Nychka, 2023 ▸). They are utilized as raw materials for synthesis or as finished products. In addition to their industrial relevance, sodium silicates in particular have been the focus of extensive research due to their intriguing physico-chemical properties, such as their high ion-exchange capacity and selectivity, as well as their two-dimensional sodium diffusion and conductivity [see the review of Kahlenberg (2010 ▸), and references cited therein]. In addition, the structural chemistry of crystalline sodium silicates presents crystallographers with a range of challenges, including polytypism, polymorphism, temperature and/or pressure-dependent phase transitions, pseudo-symmetry, complex twinning phenomena and incommensurately modulated structures (Kahlenberg, 2010 ▸). Many of these structural issues have only recently been resolved, though in some cases, they have been recognized for several decades. On the other hand, lithium silicates, such as Li_2_[SiO_3_] or Li_2_[Si_2_O_5_], are important phases in glass-ceramics (Höland & Beall, 2002 ▸). Notably, the first glass-ceramic ever developed by Stookey (1959 ▸) was based on lithium disilicate as the major crystalline phase. From an applications perspective, the silicates of the alkali metals with higher atomic numbers (Rb, Cs) have received less attention. This is a direct consequence of their extreme hygroscopicity, which precludes their direct utilization in industrial inorganic chemistry.

A limited number of rubidium silicates are known, exhibiting different forms of condensation of the [SiO_4_] tetrahedra ranging from dimers to framework structures (see Section A of the supporting information). Terminated silicate entities have been reported in sorosilicate Rb_6_[Si_2_O_7_] (Hoch & Röhr, 2001 ▸) and cyclotrisilicate Rb_6_[Si_3_O_9_] (Hoch & Röhr, 2001 ▸). A unique unbranched double tricyclosilicate group has been found in the compounds Rb_10_[Si_6_O_17_] and Rb_14_[Si_4_]­[Si_6_O_17_] (Hoffmann *et al.*, 2001 ▸; Hoffmann & Fässler, 2006 ▸). Infinite 2D silicate units are known in the phyllosilicate Rb_2_[Si_2_O_5_] (de Jong *et al.*, 1994 ▸). Furthermore, two different framework structures have been published: for Rb_6_[Si_10_O_23_] a monoclinic room-temperature form and a hexagonal high-temperature form that are both derived from the tridymite framework structure (Schichl *et al.*, 1973 ▸; Lapshin *et al.*, 2006 ▸; Huang *et al.*, 2022 ▸).

**Figure 1:**
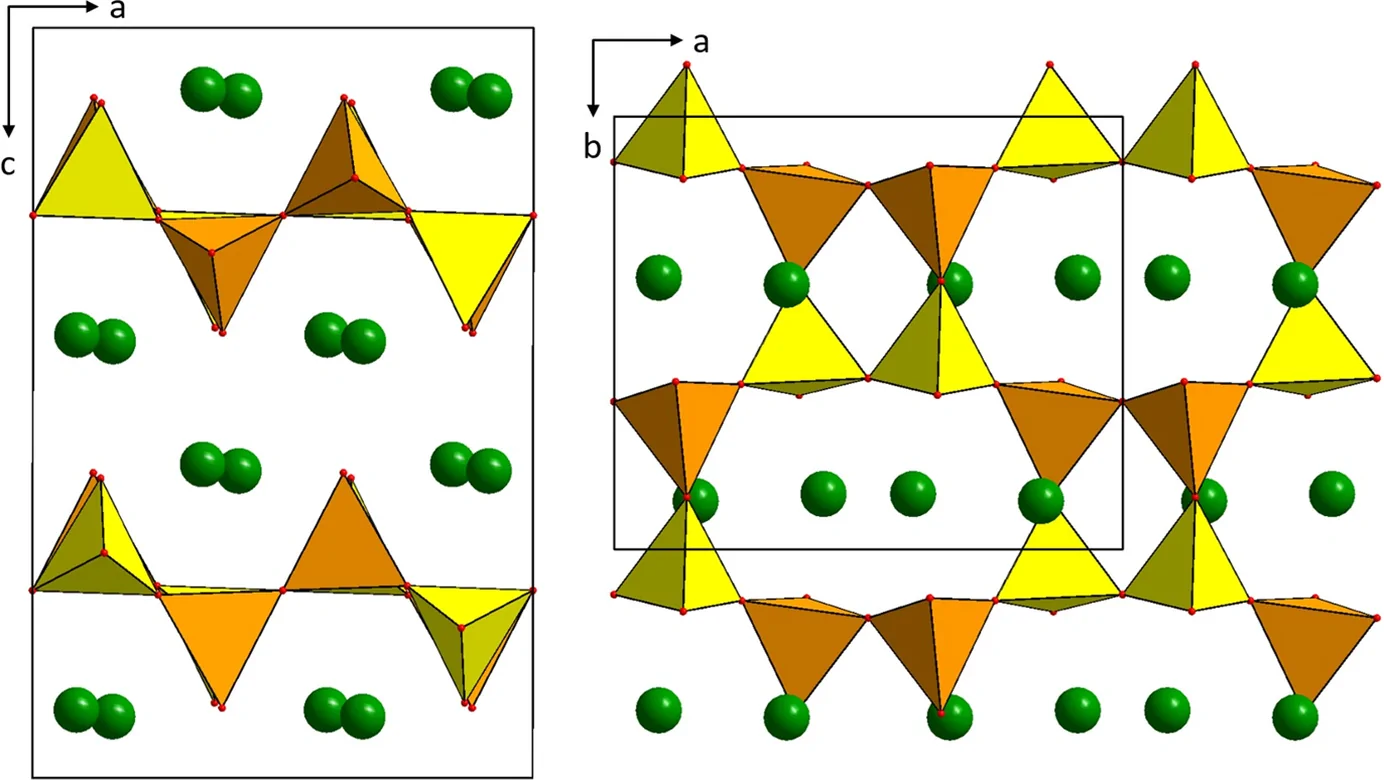
Crystal structure of Rb_2_[Si_2_O_5_] in a view along the *b* (left-hand view) and *c* (right-hand view) axes as reported by de Jong *et al.* (1994 ▸). Orange and yellow color are for tetrahedra around Si1 and Si2, respectively; Rb atoms are drawn as green spheres.

For most of the compounds mentioned above high-quality diffraction data and satisfactory crystal structure refinements have been published. However, the crystal structure of the phyllosilicate Rb_2_[Si_2_O_5_] previously posed challenges, so that even the authors presenting the data state that ‘with such high *R* factor (0.121) the structure on itself would not be publishable’ (de Jong *et al.*, 1994 ▸). They report the crystal structure as being composed of [Si_2_O_5_]^2−^ layers (Fig. 1 ▸) and intercalated Rb cations. Within the layers [SiO_4_] tetrahedra form fourfold and eightfold rings and are hence categorized as 4.8^2^ nets (Hawthorne *et al.*, 2019 ▸). Apart from unacceptable high *R* values of the presented crystal structure (de Jong *et al.*, 1994 ▸) there are several issues that demand further examination, *e.g.* unrealistically low bond valence sums (bvs) for the cations (Si1: 3.61 v.u. (v.u. for valence units), Rb2: 0.51 v.u.; calculated for Rb–O up to 3.2 Å using bond-valence parameters from Gagné & Hawthorne, 2015 ▸), and non-published temperature factors.

Rb_2_[Si_2_O_5_] was synthesized as a by-product in a study on ternary silicates with alkali and alkaline earth metals (Kahlenberg *et al.*, 2016 ▸) and found to have an incommensurately modulated crystal structure, in which several atomic positions are best described using discontinuous positional modulation functions (Petříček *et al.*, 1995 ▸; Wagner & Schönleber, 2009 ▸; Petříček *et al.*, 2016 ▸). This study aims to address the open questions associated with this crystal structure.

## 2. Experimental

### 2.1. Synthesis

The synthesis experiment for 1 g of a sample with a Rb_2_O:CaO:SiO_2_ molar ratio of 4:1:10 (or Rb_8_CaSi_10_O_25_) was based on a stoichiometric mixture of the following starting materials: Rb_2_CO_3_ (Aldrich, 99.8%), CaCO_3_ (calcite, Merck, >99.9%) and SiO_2_ (quartz, AlfaAesar, 99.995%). The platinum crucible was covered with a lid and placed in a resistance heating box furnace. The container was heated in air from room temperature to 1273 K at a rate of 2 K min^−1^. The sample was annealed at the maximum temperature for 60 min and subsequently cooled to 1073 K at a rate of 0.5 K min^−1^ before final quenching to ambient conditions.

The resulting product consisted of plate-like, colorless crystals embedded in a glassy matrix. Individual crystals reached sizes of up to several mm^3^, although smaller crystals were also observed. The crystals exhibited interference colors of predominantly first-order gray under crossed polarizers (for specimens of 20–40 µm thickness), with some of the larger crystals also showing interference colors of second order. Unexpectedly, the crystalline phase obtained was later, in the course of crystal structure determination (Section 3), identified as Rb_2_[Si_2_O_5_]. To keep the strongly hygroscopic crystals from disintegration they were stored in a closed glass vial in the dry atmosphere of a desiccator at all times.

### 2.2. Single-crystal diffraction

Single crystals taken out of the desiccator were immediately transferred to Paratone N oil (Hampton Research) and suitable crystals were selected under the polarization binocular using cross-polarized light in order to check for sharp extinction. Subsequently suitable single crystals were mounted on a loop and fixed in a dried air gas stream of 193 K generated by an Oxford Cryosystems Desktop Cooler. Single-crystal diffraction was performed on an Oxford Diffraction Gemini R Ultra diffractometer, which was equipped with a four-circle kappa goniometer and a Ruby CCD detector. The data were processed using the *CrysAlis PRO* software package (version 1.171.44) (Rigaku Oxford Diffraction, 2020 ▸). Following indexing, the diffraction pattern was integrated. The data reduction process involved Lorentz and polarization corrections. Data were analytically corrected for absorption using 16 indexed faces. For solution of the crystal structure the program *Superflip* (Palatinus, 2004 ▸) was used, crystal structure refinement was performed using the program *Jana2020* (version 2.1 29/08/2025) (Petříček *et al.*, 2023 ▸). For experimental details see Table 1 ▸.

### 2.3. Indexing

Reflections of high intensity only (*i.e.I*/σ > 30) can be indexed with small integer indices *hkl* to a *C*-centered lattice with *a*_av_ ≈ 9.87, *b*_av_ ≈ 8.40, *c*_av_ ≈ 14.77 Å, α = γ = 90°, β ≈ 90.1°. However, a number of additional reflections not accounted for by such a unit cell and with considerably weaker intensities are present along the b* direction.

**Figure 2:**
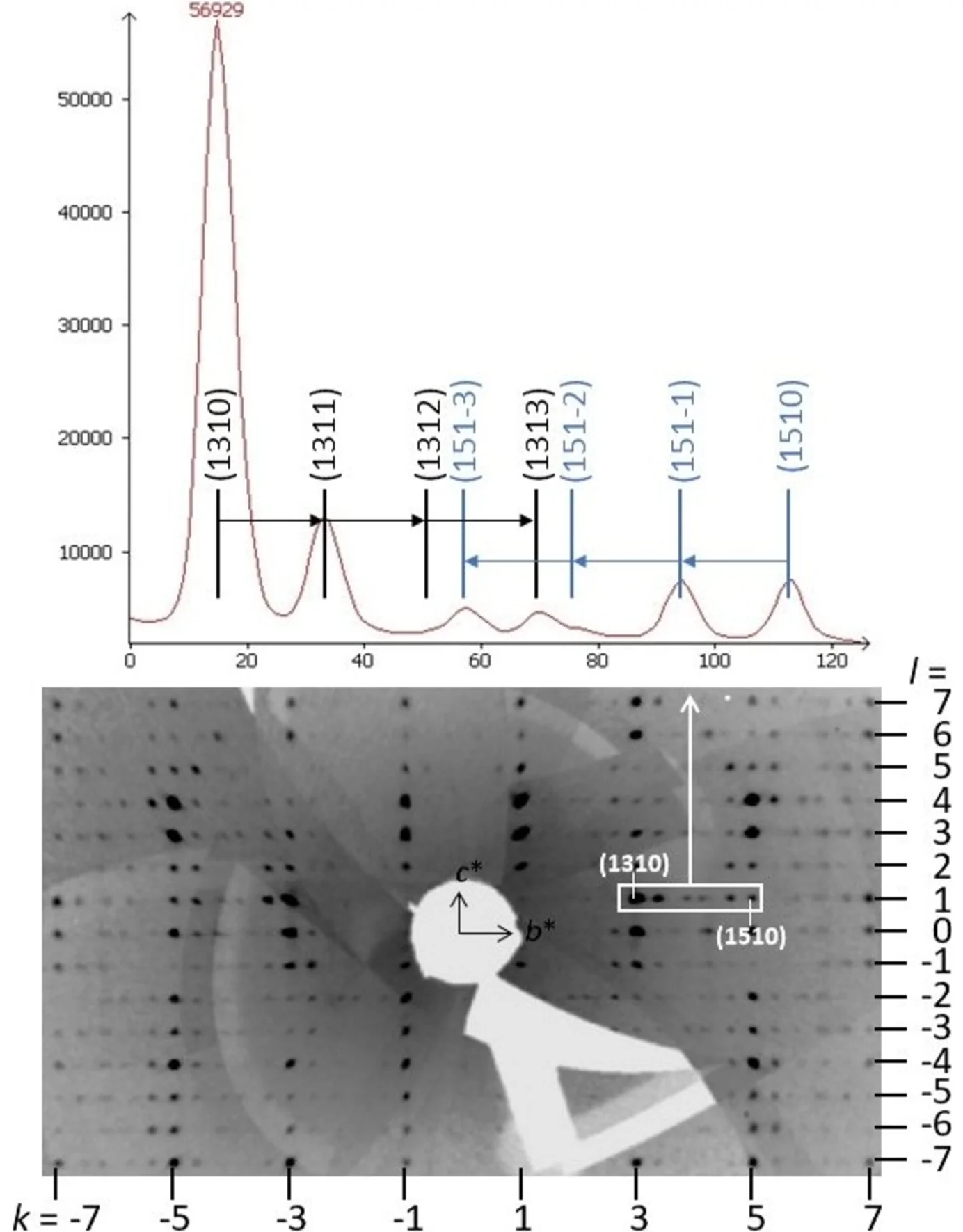
(Bottom view) Section of the (1*kl*) layer to highlight the position of satellite reflections 1*klm* relative to main reflections 1*kl*0. (Top view) 1D profile through reflections in the section highlighted in the white rectangle. Main reflections are present for *h*+*k* = 2*n*. Satellite reflections seem to separate the space between two present main reflections along b* into five equal divisions. However, close examination shows the divisions are not equal. Rather, the observed reflections are interpreted as clearly distinguishable satellite reflections of first order, *hkl ± *1, adjacent to main reflections. Satellite reflections of second and third order, *hkl ± *2 and *hkl ± *3, have weaker intensities. Note that their positions are rather close and partially overlap.

A 1D profile along the b* direction (Fig. 2 ▸) reveals the position of several satellite reflections, whereupon the strongest intensities can be found for satellite reflections with *k* ≈ *n* ± 0.38; these reflections are clearly distinguishable from the main reflections and higher order satellite reflections with very faint intensities. These latter reflections arise from an overlay of reflections with *k* ≈ *n* ± 2 × 0.38 and *n* ± 3 × 0.38. All reflections with *k* ≠ *n* values are hence regarded as satellite reflections of an incommensurately modulated crystal structure with unit-cell parameters *a* = 9.8695 (6), *b* = 8.4012 (6), *c* = 14.7701 (10) Å, β = 90.113 (5)°, *V* = 1224.7 (1) Å^3^, and a modulation wavevector **q** = 0.377 (1)b*.

The presence of satellite reflections was monitored with increasing temperature by a preliminary screening without full data collection. All main and satellite reflections were present up to 288 K, thus indicating that the incommensurately modulated crystal structure remains up to room temperature.

## 3. Refinement

### 3.1. Average three-dimensionally periodic crystal structure

The average crystal structure was solved from the main reflections *hkl*0 in space group *C*2/*c* (No. 15); for a list of atomic coordinates see supporting information (Section B). All parameters of the average three-dimensionally periodic crystal structure will be indicated with the subscript av. Due to the monoclinic angle being very close to 90° special care was taken to exclude the possibility of twinning: (i) There was no indication of split reflections and (ii) a refinement of the final structure model with a twofold twin axis along the *c* direction did not improve the results compared to the refinement without a twin model. Furthermore, a refinement of the twin fractions gave values of 1 and 0 for the two twin individuals, respectively.

**Figure 3:**
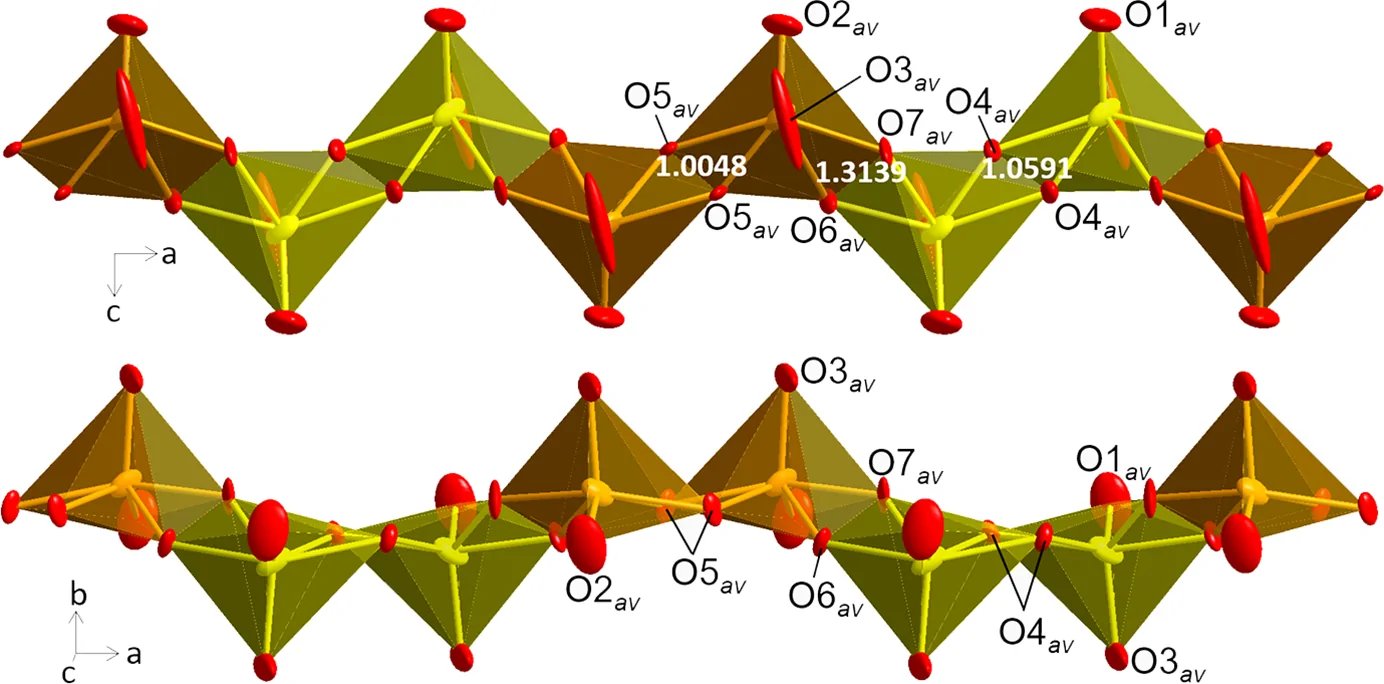
Selected section of the three-dimensionally periodic average crystal structure of Rb_2_[Si_2_O_5_] refined from main reflections *hkl*0, that is disregarding scattering information from the satellite reflections* hklm*. Top view along the *b* axis. Bottom view approximately along the *c* axis. Yellow and orange colors for polyhedra around Si1_av_ and Si2_av_ respectively; displacement ellipsoids for oxygen atoms (red) are shown at the 50% probability level. O4_av_–O4_av_, O5_av_–O5_av_ and O6_av_–O7_av_ have an occupancy of 0.5 and have to be considered as split atom position with a disordered arrangement. With this assumption [SiO_4_] tetrahedra share corners to form chains along the *a* direction.

In the structure solution two symmetrically independent rubidium, two silicon and seven oxygen positions were found. However, the apparent sixfold coordination of silicon atoms at distances ranging from ∼1.49 to 1.79 Å (Table 2 ▸) can in this approach only be interpreted by the simultaneous presence of two distinct [SiO_4_] tetrahedra with a disordered arrangement. [SiO_4_] polyhedra are connected to form (i) chains along the *a* direction (Fig. 3 ▸) and (ii) chains along the *b* direction that intersect each other to form corrugated sheets in the *ab* plane; adjacent layers are shifted relative to each other by ± *b*/4.

Rubidium atoms are located between the silicate layers. While bond-valence-sum analysis is only indicative – particularly for disordered structures and in the presence of large anisotropic displacement parameters (ADPs) in the average structure – and should therefore be interpreted with caution, it still provides a useful guide. Notably, when Rb–O contacts are considered up to 3.20 Å (see Table 2 ▸), Rb1 and Rb2 adopt [6]- and [8]-coordination, respectively; yet their bvs values, calculated with the parameters of Gagné & Hawthorne (2015 ▸), remain significantly below the expected valence of +1. This points to pronounced underbonding, particularly for Rb1.

Each silicon atom is coordinated by O1_av_ and O2_av_ in the polyhedra of Si1_av_ and Si2_av_, respectively, bonding to Rb, whereas the remaining coordinating oxygen atoms are shared with adjacent silicon polyhedra. Displacement ellipsoids representing ADPs of O1_av_ and O2_av_ are somewhat enlarged in comparison to *e.g.* O4_av_–O7_av_ and have an oblate form. The oxygen atom O3_av_ is shared by the silicon polyhedra around Si1_av_ and Si2_av_, connecting them in the *b* direction. Note that the ADPs of this O3_av_ atom have a very strongly elongated form with the long principal axis of the displacement ellipsoid almost parallel and only slightly inclined to the *c* direction. The other oxygen positions, O4_av_–O7_av_, have to be interpreted as split atom positions due to their close proximity to each other (Fig. 3 ▸). Occupancy of split oxygen positions was initially set to 0.5. A refinement of the occupancy showed only minor deviation in the third decimal place, so that it was fixed to 0.5. Taking this into account, the chemical formula of the found crystal structure is Rb_2_[Si_2_O_5_], with *Z* = 8 for the unit-cell parameters given above.

**Figure 4:**
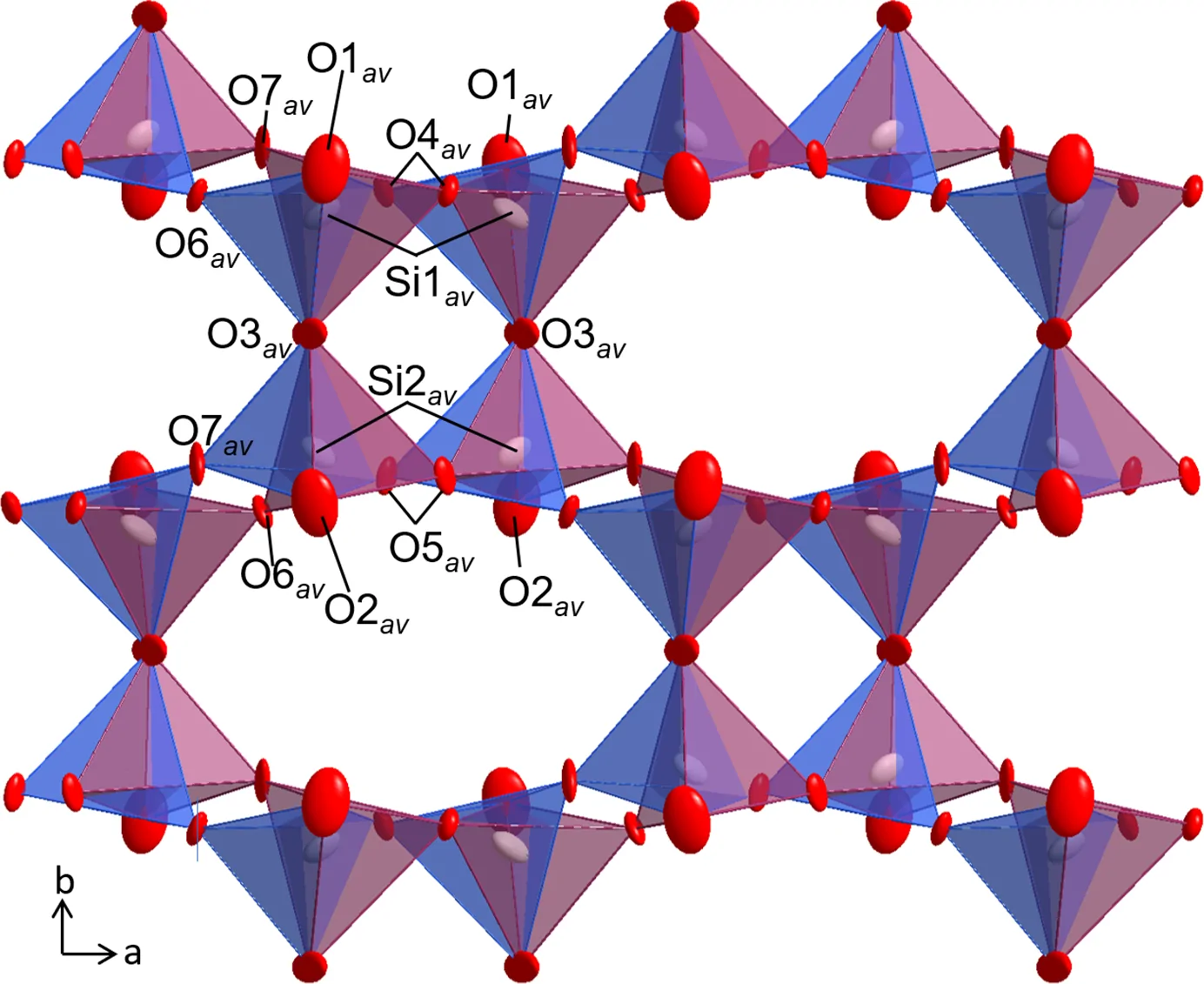
Each [SiO_4_] tetrahedron can occupy one of two different possible orientations, A or B, that are drawn in purple and blue color, respectively. The orientation of any tetrahedron within a chain determines the orientation of all other tetrahedra adjacent along the *a* axis. Displacement ellipsoids for oxygen (red) and rubidium atoms (green) are shown at the 50% probability level.

The coordination around silicon atoms (Fig. 4 ▸) can now be interpreted as [SiO_4_] tetrahedra with two different orientations, *A* or *B*, that are related to each other by either twofold rotation or 2_1_ screw axes running parallel to the *b* direction within the silicate layer. Notably, oxygen atoms O4_av_ and O5_av_ belonging to tetrahedra in the *A* or *B* orientation are each symmetry-related by a twofold axis. Furthermore, while their *x* and *z* coordinates are almost identical, their *y* coordinate differs by ≈0.5. O6_av_ and O7_av_ atoms of one or the other tetrahedral orientation are symmetry-related by a 2_1_ screw axis. These observations, together with the overall arrangement of the [SiO_4_] tetrahedra, indicate positional disorder about a mirror plane that is absent in the average structure; this putative mirror plane would be situated in the *ac* plane and pass through O3_av_. A *c* glide plane perpendicular to *b* relates atomic positions to their symmetry-equivalent positions in the adjacent silicate layers.

A refinement of the average crystal structure model using ADPs for all atoms yields a final *R*_1_ value of 0.039 (Table 1 ▸). These results are quite reasonable, considering the lack of information from the satellite reflections to calculate the positional modulation of the atomic positions. Moreover, it is reasonable to assume that the modulation is primarily affecting atomic positions of silicon and oxygen atoms and the heavy Rb atom to a much lesser extend. However, several aspects remain unsatisfactory: (i) Significant distortion of the [SiO_4_] tetrahedra, resulting in unacceptably short and long interatomic distances (Table 2 ▸). (ii) Relatively large ADPs with strongly prolate displacement ellipsoids for oxygen atoms O1_av_ to O3_av_. (iii) An incomplete description of the split oxygen positions O4_av_ to O7_av_. (iv) Bond valence sums for the rubidium atoms that are much too low.

### 3.2. Incommensurately modulated crystal structure in (3 + 1)-dimensional superspace

In order to overcome the crystal-chemical problems concerning the average three-dimensionally periodic structure model mentioned above, satellite reflections of first order were included in the intensity integration and subsequent structural refinement process for the (3 + 1)-dimensional crystal structure model. For *C*2/*c* and modulation direction [001] there are two superspace groups possible: *C*2/*c*(0β0)00 or *C*2/*c*(0β0)*s*0 of which the latter was found to match the observed reflections. This corresponds to superspace group 15.3 *B*2/*b*(00γ)*s*0 (Janssen *et al.*, 2006 ▸) with *c* as the monoclinic axis. Second and third-order reflections, although present, could not be integrated satisfactorily. Only ∼19% of all integrated *hkl*3 reflections were considered observed with *I*/σ > 3. *R*_int_ of all *hkl*3 reflections was 0.3119. Including these reflections did not improve the crystal structure refinement. On the contrary, such a refinement resulted in non-positive definite ADP-parameters of several atoms. Therefore, *hkl*2 reflections and *hkl*3 reflections were not used.

In a first attempt the split oxygen atom positions O4_av_–O7_av_ of the average structure were modeled using a crenel function, (Petříček *et al.*, 1995 ▸; Petříček *et al.*, 2016 ▸), to define the occupational modulation. For all other atom positions harmonic modulation wavefunctions were used. The crenel function is periodic but discontinuous, defined as 1 when the atomic position is occupied and 0 when it is unoccupied. The discontinuity in occupation indicates that the atom exists only within a fraction Δ of the full interval of *x*_4_, specifically within the interval (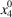 − Δ/2, 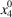 + Δ/2), where Δ < 1; in this case the width Δ of the crenel function for all atoms was defined as 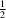. This, however, did not result in an acceptable structure model due to non-realistic interatomic Si–O distances and some non-positive definite ADP tensors.

As Si1*a*, O1, O3, O4*a* and O5*a* (configuration 1) and Si1*b*, O1, O3, O4*b* and O5*b* (configuration 2) belong each to one of two mutually exclusive configuration of the tetrahedra, restrictions have to be employed, to make sure that only one of the two possible configurations are present in any *t* section. This is done by introducing a shift of 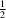 between the *t*_0_ of the respective atoms, *e.g.* the following equation for Si1*a* and Si1*b*:
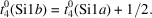
The corresponding equations used are given in the supporting information (Section C). This ensures that for all physical space sections only atoms belonging to one of the alternative tetrahedral positions are present.

**Figure 5:**
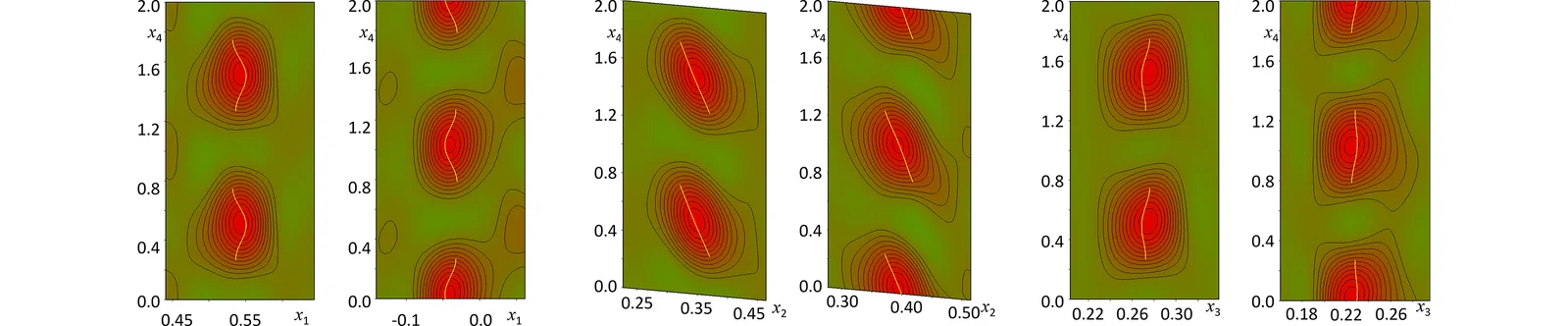
Fit of the atomic modulation functions of the O4*a* and O4*b* atoms (yellow line) to the electron density. *x*_1_–*x*_4_, *x*_2_–*x*_4_, and *x_3_*–*x_4_* maps, intersecting the four dimensional *F*_obs_ Fourier synthesis at *x*_1_ = 0.5427, *x*_2_ = 0.3453, *x*_3_ = 0.2716 for O4*a* and *x*_1_ = −0.0392, *x*_2_ = 0.3965, *x*_3_ = 0.2283 for O4*b*, respectively. Contour lines from 2 to 20 e Å^−1^ in 2 e Å^−1^ intervals. The maps are summed up over 1 Å in the secondary directions.

The atomic positions Si1*a*, Si1*b*, Si2a, Si2b, O4*a*, O4*b*, O5*a* and O5*b* were each described using a crenel function in combination with one additional positional modulation wave, that has been realized with the functions of x-harmonics with two coefficients in the crenel interval as described by Petříček *et al.*, 2016 ▸. To model the O1, O2 and O3 atoms the use of continuous positional wavefunctions provided better results compared to the use of a crenel function. O1 and O2 were modeled using modulation functions with one harmonic, for O3 a positional modulation wave with two harmonics were used. Positional modulation was also used for Rb1 and Rb2, however, in addition a modulation of the ADP parameters has been employed for this heavy element. A list of the atomic positions is given in the supporting information (Section C), for interatomic distances see Tables 3 ▸ and 4 ▸. To exemplify the positional modulation of the atoms modeled with a crenel function Fig. 5 ▸ shows the fit of the atomic modulation functions of the O4*a* and O4*b* atoms to the electron density using 2D sections *x*_1_–*x*_4_, *x*_2_–*x*_4_, *x*_3_–*x*_4_ of the (3 + 1)-dimensional electron density. For the respective images of all other atoms see supporting information (Section C).

## 5. Discussion

As described by de Jong *et al.* (1994 ▸), and discussed in the refinement of the average crystal structure above, Rb_2_[Si_2_O_5_] is composed of puckered [Si_2_O_5_]^2−^ layers perpendicular to the *c* axis / parallel to the *ab* plane, with adjacent layers being shifted by  ±  *b*/4, and rubidium atoms in-between.

**Figure 6:**
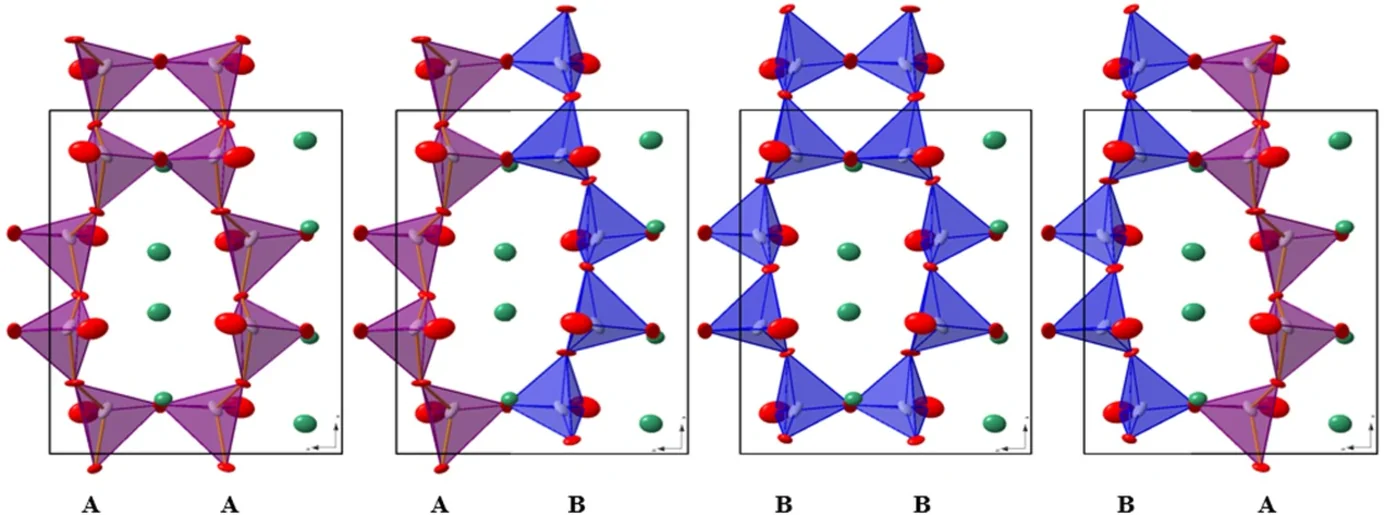
Four different combinations of adjacent chains and their orientational pattern. Chains in the *A* orientation in purple, *B* orientation in blue color. Displacement ellipsoids for oxygen (red) and rubidium atoms (green) are shown at the 50% probability level.

Within the [Si_2_O_5_]^2−^ layers tetrahedra are connected via oxygen atoms to form chains running parallel to the *a* axis with a periodicity of four tetrahedra, so called *vierer* single chains (Liebau, 1985 ▸); these chains are connected to form layers with fourfold and eightfold rings of [SiO_4_] tetrahedra. Since the chains parallel to the *a* axis are composed of [SiO_4_] tetrahedra, each of which can adopt one of two possible orientations, *A* or *B*, the orientation of any tetrahedron within a chain determines the orientation of all other tetrahedra adjacent along the *a* axis. However, for the neighboring chain, which is adjacent in the *b* direction, the [SiO_4_] tetrahedra can again adopt only one of the two possible orientation (Fig. 6 ▸).

**Figure 7:**
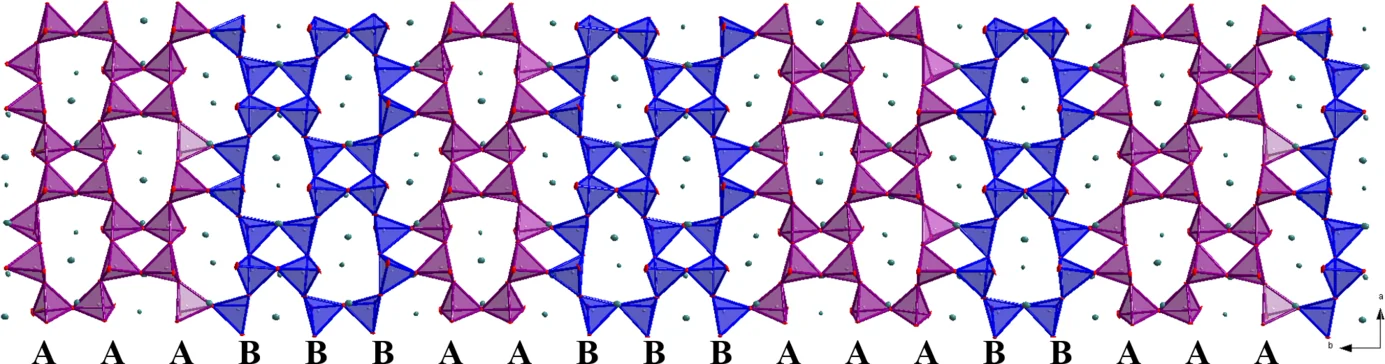
Approximante structure of 2 × 0.5 × 10 unit cells showing the [Si_2_O_5_] layer. Blue and purple color for the [SiO_4_] tetrahedra display the two different orientations *A* and *B*, respectively, green spheres: Rb. Notably, two or three chains are of the same orientation, then the orientation switches. Furthermore, four- and eightfold rings between adjacent chains have a different configuration between neighboring *AA*, *AB*, *BB* and *BA* chains. Displacement ellipsoids for oxygen (red) and rubidium atoms (green) are shown at the 50% probability level.

A physical space section (Fig. 7 ▸) reveals that groups of two or three adjacent chains are composed of tetrahedra in the *A* orientation, followed by a switch to the *B* orientation. The sequence of orientations along the *b* direction is described as *A*-*A*-*A*-*B*-*B*-*B*-*A*-*A*-*B*-*B*-*B*-*A*-*A*-*A*-*B*-*B* or, alternatively, as a sequence of 3-3-2-3-3-2 chains with equivalent orientational patterns. However, focusing on such a small scale the found 8*n*-fold repeat pattern is identical for **q** = 0.377 (1)b* and for **q** = 0.375b* = 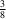
b*. Instead, 8(0.377 − 0.375) = 0.016*n* deviations from the above given sequence appear over *n* chains, *i.e.* one to two deviations per 100 chains.

Alternatively, the layers can be also be described by chains of [SiO_4_] tetrahedra running parallel to the *b* axis, again with a periodicity of four tetrahedra, an approach less convenient for the description of the positional modulation.

**Figure 8:**
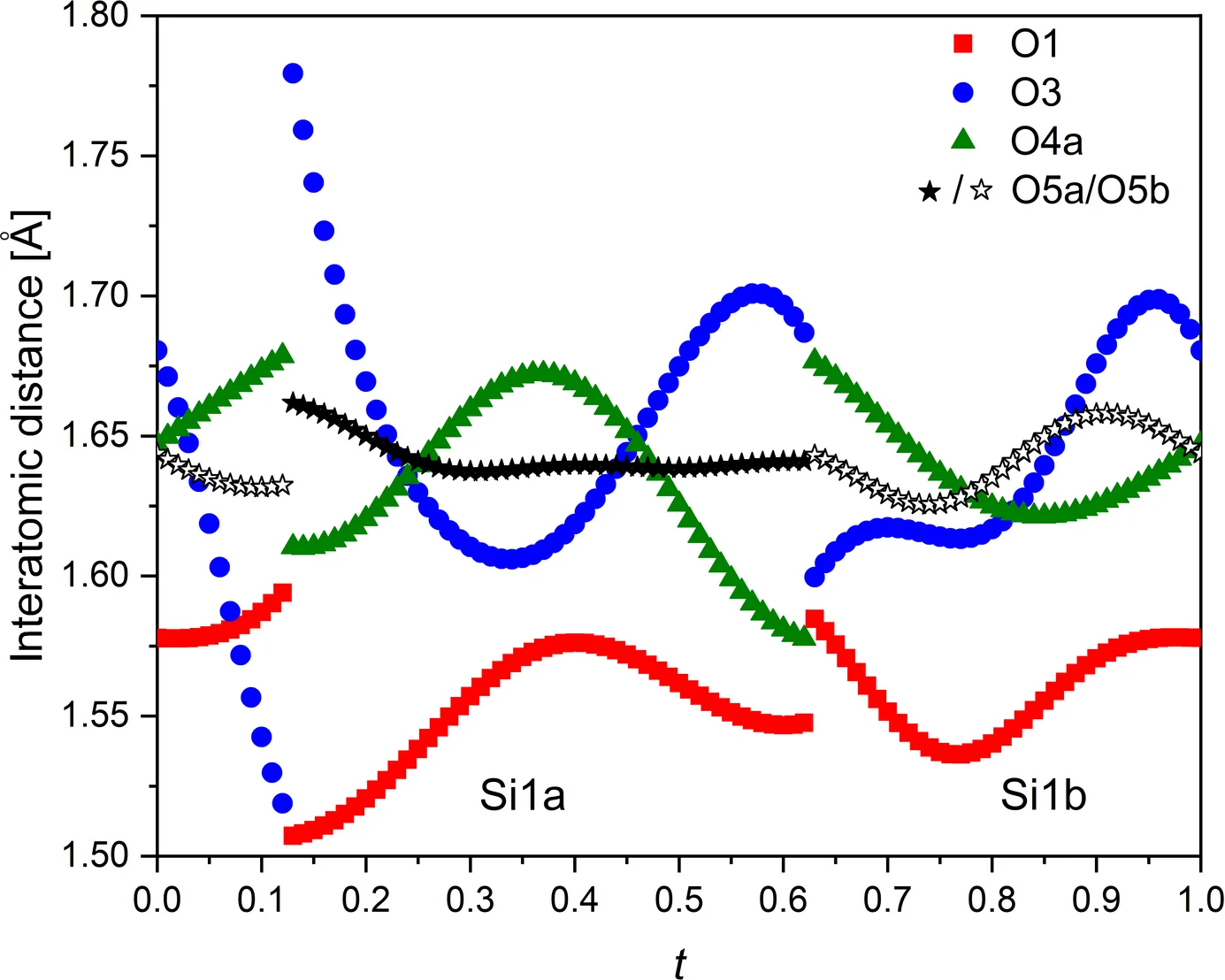
Interatomic Si1–O distances as a function of phase *t* for the two different orientations of the [Si1O_4_] tetrahedra.

Interatomic Si–O distances range from 1.49 (2) to 1.79 (2) Å (Table 3 ▸, Figs. 8 ▸ and 9 ▸), which is unacceptable with respect to commonly known silicate phases (Liebau, 1985 ▸). However, close examination of interatomic distances as a function of *t* reveal that the most objectionable values are found around the positions in *t*, where the two orientations switch, i.e around *t* = 0.13 and *t* = 0.63, whereas across most of the crystal structure the Si–O distances are closer to chemically reasonable values. The most extreme values, *e.g.* Si1—O3 around *t* = 0.13 (Fig. 8 ▸), have to be considered as Fourier truncation effects due to the lack of scattering information from the satellite reflection of second and third order, that where not used in the refinement.

The pronounced spread of Si–O distances in Rb_2_[Si_2_O_5_] is in accordance with the observation that in alkali metasilicates the tetrahedral distortion increases with increasing condensation of the [SiO_4_] tetrahedra (Hoch & Röhr, 2001 ▸). Similar values have been observed for other alkali phyllosilicates, *i.e.A*_2_Si_2_O_5_ with *A* = Li, Na, K, Rb, Cs. All of these compounds contain four-, six-, eight- and tenfold rings within the silicate layers, which exhibit several different patterns of corrugation or undulation (de Jong *et al.*, 1994 ▸; de Jong *et al.*, 1998 ▸; Kahlenberg *et al.*, 1999 ▸; Hoch & Röhr, 2001 ▸).

**Figure 10:**
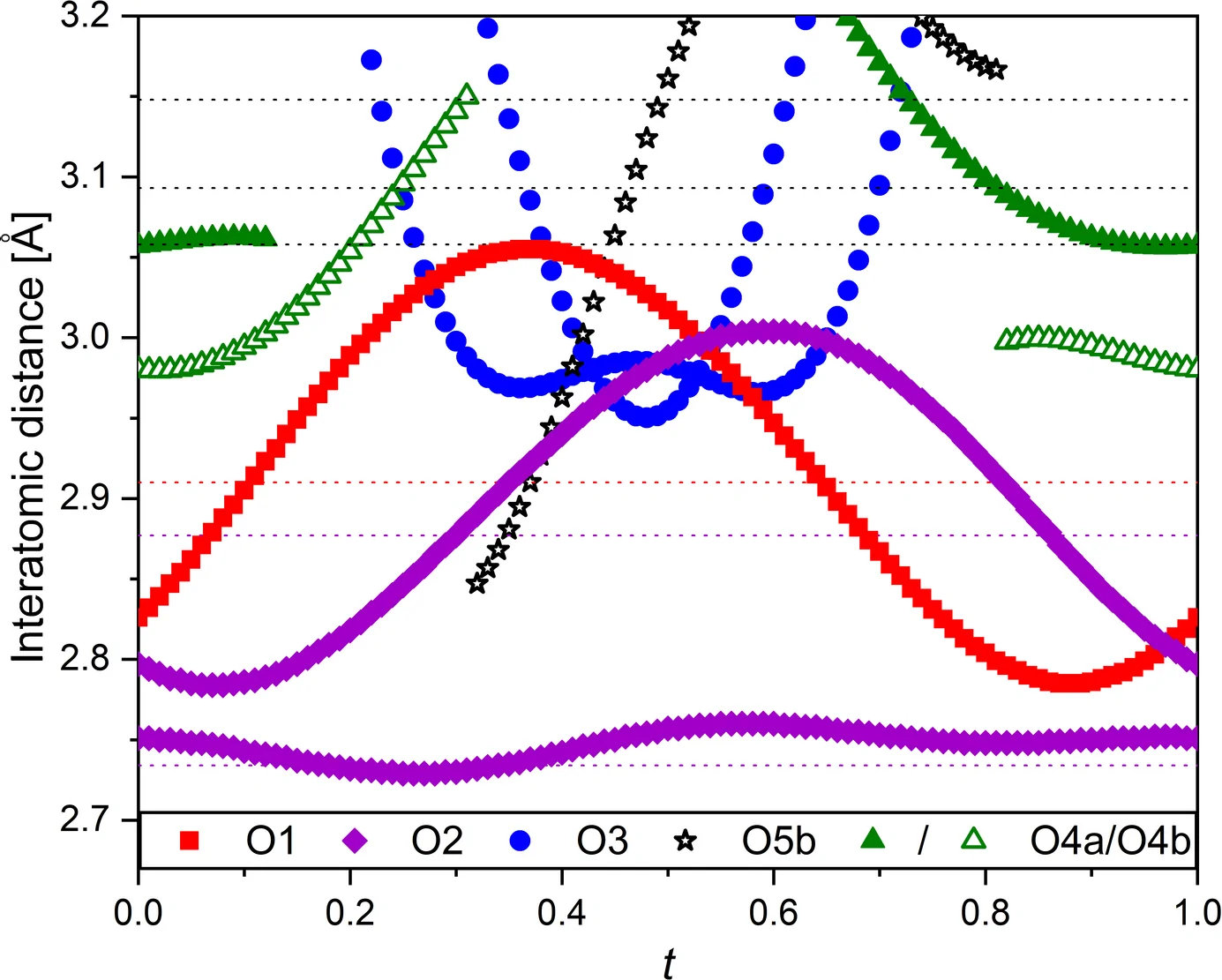
Interatomic Rb1–O distances and bond valence sums for Rb1 in the modulated crystal structure of Rb_2_[Si_2_O_5_] as a function of *t*, for comparison interatomic Rb–O_av_ distances of the average three-dimensionally periodic structure are inserted as dotted lines.

The oxygen atoms nearest to the rubidium atoms (Table 4 ▸) are the terminal oxygen atoms of the [SiO_4_] tetrahedral chains, *i.e.* O1 and O2 (Figs. 10 ▸ and 11 ▸). Although other oxygen atoms also contribute to the coordination of the Rb atoms, it is especially the close oxygen atoms with the highest contribution to the bvs values that have shorter interatomic distances to Rb in the modulated crystal structure in comparison to the average structure. Therefore, the bvs are more balanced and closer to the expected value of +1, ranging from 0.9384 (2) to 1.2390 (2) v. u. when calculated for distances up to 3.2 Å (Figs. 10 ▸ and 11 ▸, Table 4 ▸), compared to those in the average structure. Both rubidium positions are [6]-fold coordinated in most parts of the crystal structure, although [5]- and [7]-coordination is also possible.

**Figure 12:**
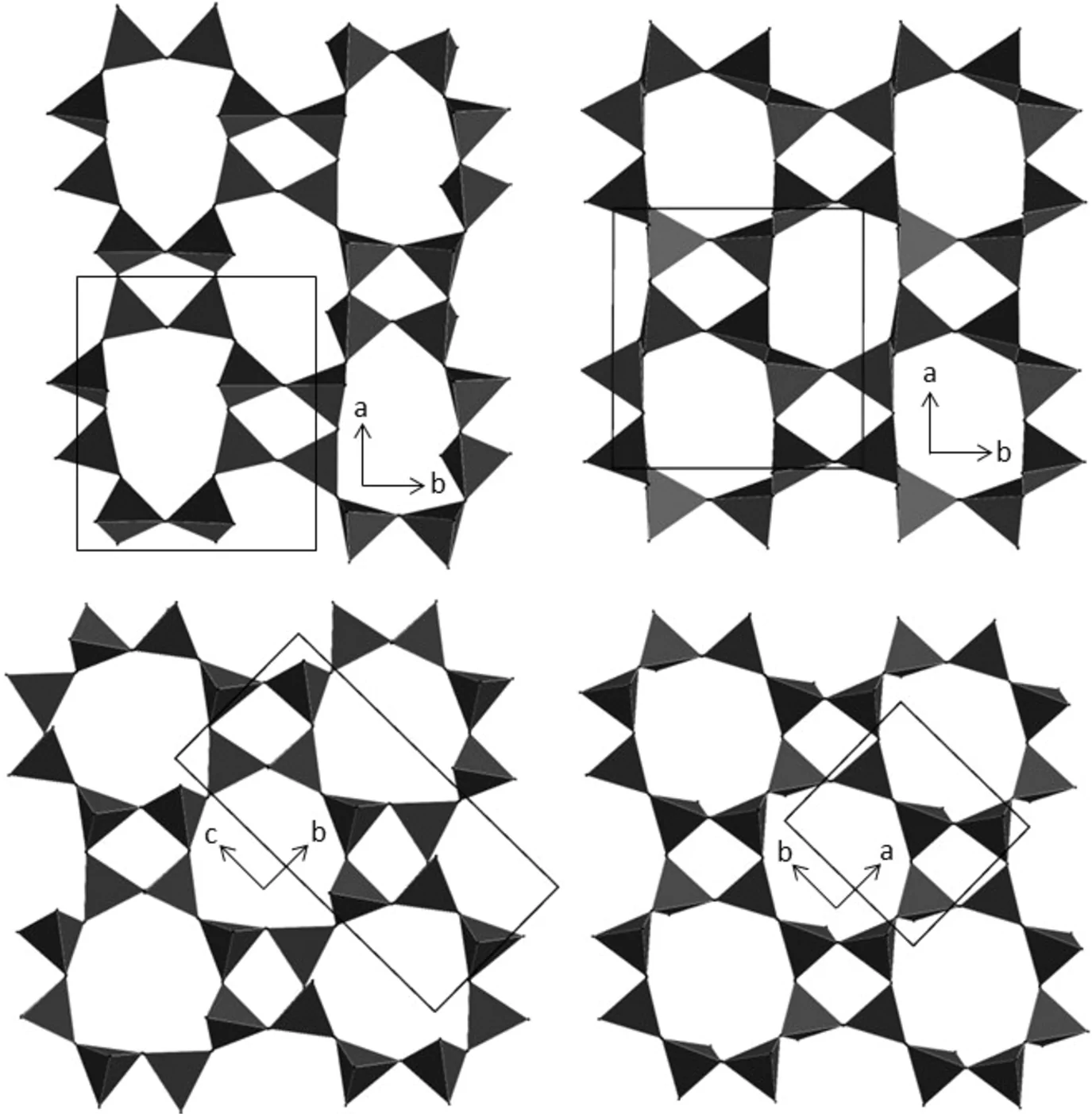
4.8^2^ net in the silicon layers of published crystal structures. Top left: Rb_2_[Si_2_O_5_] this study. Top right: Cs_2_[Si_2_O_5_] (de Jong *et al.*, 1994 ▸). Bottom left: mountainite, KNa_2_Ca_2_[Si_8_O_19_(OH)]·6H_2_O (Zubkova *et al.*, 2009 ▸). Bottom right: cryptophyllite, K_2_Ca[Si_4_O_10_]·5H_2_O (Zubkova *et al.*, 2010 ▸).

The [Si_2_O_5_]^2−^-layer configuration observed in Rb_2_[Si_2_O_5_] is also found in Cs_2_[Si_2_O_5_] (de Jong *et al.*, 1994 ▸). Due to the larger ionic radius of Cs (1.67 Å for ^[6]^Cs^1+^), in comparison to Rb [1.52 Å for ^[6]^Rb^1+^ (Shannon, 1976 ▸)], the silicon layer in Cs_2_[Si_2_O_5_] seems to exhibit fewer distortions (Fig. 12 ▸). It may be worthwhile to investigate whether the diffraction pattern of Cs_2_[Si_2_O_5_] also shows satellite reflections, which were not observed in 1994, when area detectors were not as widely available as they are today.

In addition to Rb_2_[Si_2_O_5_] and Cs_2_[Si_2_O_5_], there are several phyllosilicates whose single layers can be categorized as [4.8]^2^ nets (Hawthorne *et al.*, 2019 ▸). Examples include minerals from the apophyllite group, however, the configuration and arrangement of the four- and eight-membered rings in these minerals differ, and their layers exhibit a different up-down (u-d) configuration of the [SiO_4_] tetrahedra. Only the silicon layers (Fig. 12 ▸) in the minerals mountainite, KNa_2_Ca_2_[Si_8_O_19_(OH)]·6H_2_O, shlykovite, KNa_2_Ca_2_[Si_8_O_19_(OH)]·6H_2_O, and cryptophyllite, K_2_Ca[Si_4_O_10_]·5H_2_O, are truly comparable (Zubkova *et al.*, 2009 ▸; Zubkova *et al.*, 2010 ▸) with the one in Rb_2_[Si_2_O_5_]. However, these layers partially contain [SiO_3_(OH)] tetrahedra, which result in a slightly larger unit mesh. Additionally, these minerals feature larger structural units between the layers.

To give an example. In mountainite, the cations Na^+^, K^+^, and Ca^2+^ all have smaller ionic radii than Rb^+^ or Cs^+^. However, each unit mesh of the silicate layer in mountainite has to fit the interlayer content of approximately 1.25 cations and 1.5H_2_O molecules, which together occupy more space than two rubidium atoms. For all of the aforementioned compounds, the unit mesh of the silicate layer can be approximately calculated as the product of the unit-cell parameters that span the plane of the silicate layer and provides a simplified measure of the layer’s mesh size. Among these compounds, the unit mesh is smallest in Rb_2_[Si_2_O_5_] (Table 5 ▸).

This observation suggests that the incommensurate modulation in Rb_2_[Si_2_O_5_] is a mechanism by which the silicate layer adapts to the relatively small size of rubidium. An undulation and/or corrugation of the silicon layer alone appears insufficient to accommodate this size mismatch. Further evidence supporting this interpretation can be found in the shape of the four- and eight-membered rings within the layers. As shown in Fig. 12 ▸, the corresponding rings in the mountainite-group minerals are larger and more circular compared to those in Rb_2_[Si_2_O_5_].

## 6. Concluding remarks

The presented crystal structure refinements of Rb_2_[Si_2_O_5_], both models—the average three-dimensionally periodic model with split oxygen atom positions as well as the (3 + 1)-dimensional superstructure model—represent a significant improvement over the crystal structure published by de Jong *et al.* (1994 ▸). All atomic parameters, particularly the anisotropic displacement parameters and bond valence sum values, fall within acceptable ranges. Additionally, the *R*_1_ values obtained for the refinements—0.0371 for the average three-dimensionally periodic model and 0.0419 for the (3 + 1) superstructure model—are markedly lower than the *R* value of 0.12 reported by de Jong *et al.* (1994 ▸).

When comparing the average three-dimensionally periodic model to the (3 + 1)-superstructure model, the latter demonstrates several advantages, including more realistic Si–O interatomic distances, more balanced bvs values for the rubidium cations, and improved shapes of the ADPs. This is explained by the fact that a description of the [SiO_4_] tetrahedra by an occupational modulation using a crenel function is superior to the occupational pattern of split atom positions in the average three-dimensionally periodic model.

Further investigations, particularly *in situ* high-temperature structural studies, could clarify the mechanism of the modulation and identify possible phase transitions, such as an incommensurate-to-normal transition (*i.e.* loss of the incommensurate modulation on heating into the unmodulated, three-dimensionally periodic basic structure described by a conventional space group), although such experiments are difficult because of the material’s extreme hygroscopicity. Furthermore, it could be advisable to reinvestigate the crystal structures of other *A*_2_[Si_2_O_5_] compounds, *e.g.* Cs_2_[Si_2_O_5_], that have previously been published with unsatisfactory structural parameters (de Jong *et al.*, 1994 ▸).

## Supplementary Material


8poePc7qaeg

CCDC reference: 2580078

## Figures and Tables

**Figure 9:**
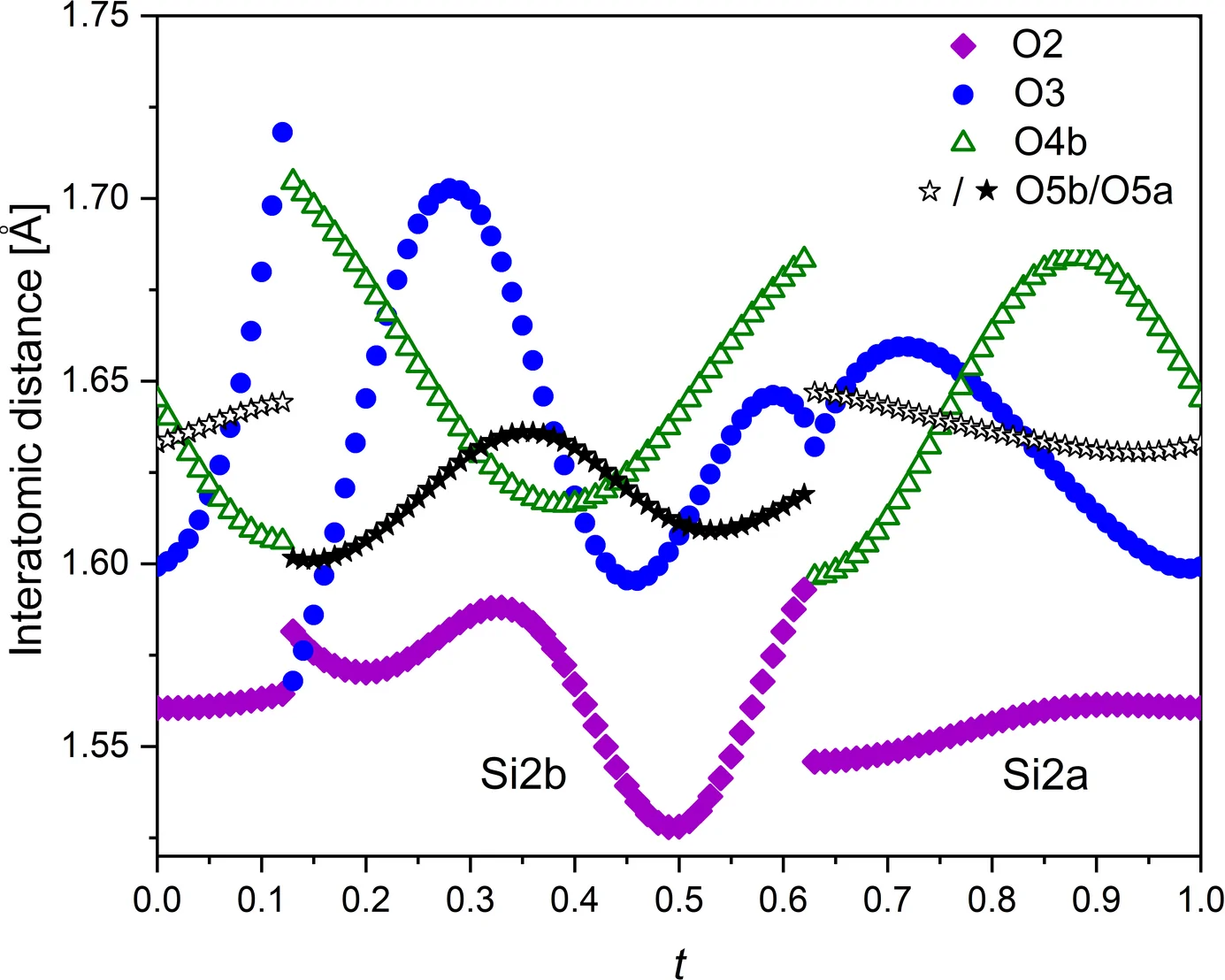
Interatomic Si2–O distances as a function of phase *t* for the two different orientations of the [Si2O_4_] tetrahedra.

**Figure 11:**
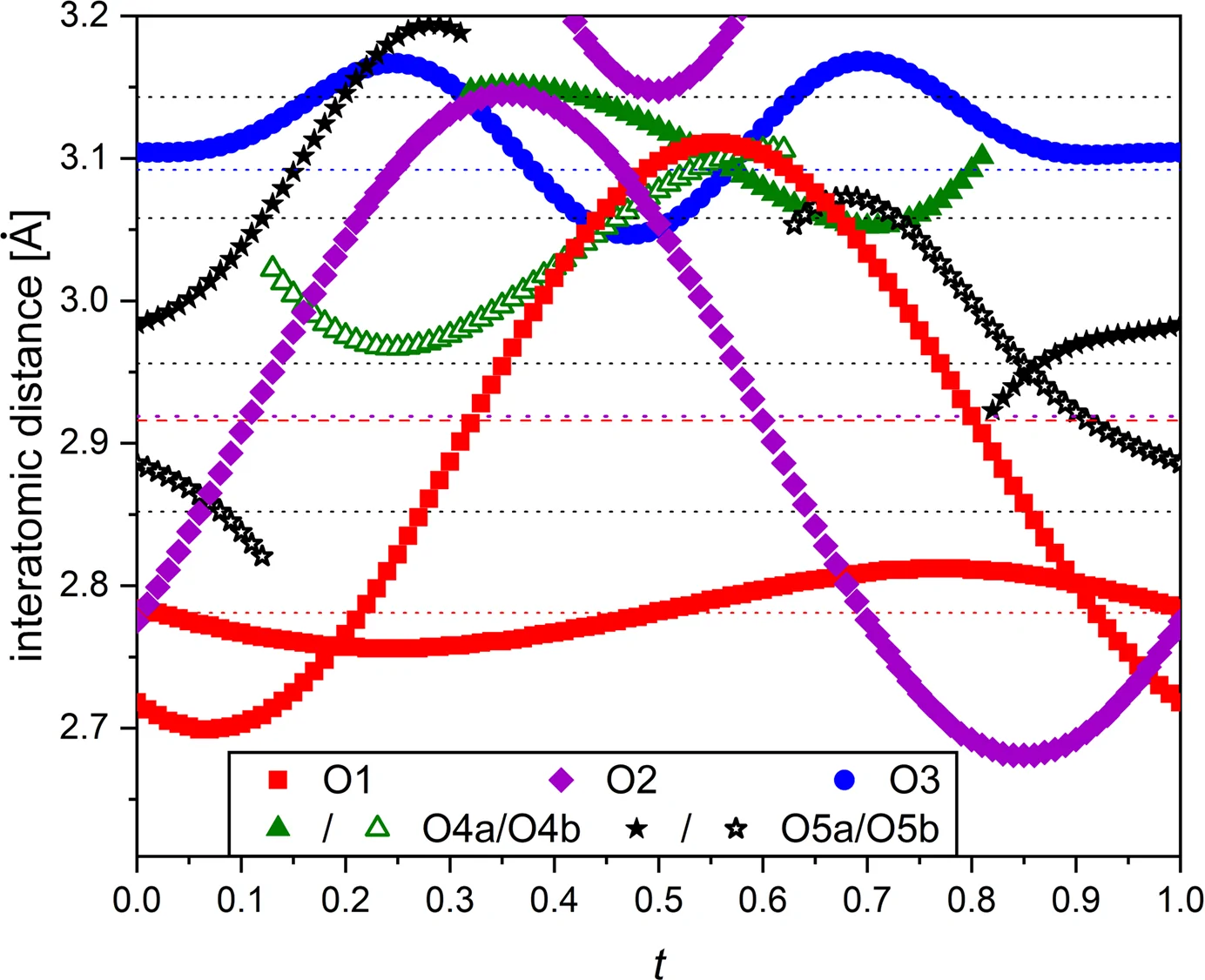
Interatomic Rb2–O distances and bond valence sums for Rb2 in the modulated crystal structure of Rb_2_[Si_2_O_5_] as a function of *t*; for comparison interatomic Rb–O_av_ distances of the average three-dimensionally periodic structure are inserted as dotted lines.

**Table 1:** Experimental details for Rb_2_[Si_2_O_5_]

	Average three-dimensional periodic	Incommensurately modulated (3 + 1)
Diffractometer	Rigaku Oxford Diffraction Gemini	
Mo *K*α, wavelength λ (Å)	0.71073	0.71073
Monochromator	Graphite	Graphite
Detector	Ruby CC	Ruby CC
Temperature (K)	193	193
*a*, *b*, *c* (Å)	9.8662 (6), 8.3986 (5), 14.7641 (9)	
β (°)	90.114 (5)	90.114 (5)
Modulation wavevector	–	[0, 0.377 (1), 0]
*V* (Å^3^)	1223.4 (1)	1223.4 (1)
*Z*	8	8
*D*_*x*_ (g cm^−3^)	3.3347	3.3347
Space/superspace group	*C*2/*c* (No. 15)	*C*2/*c*(0β0)*s*0 (No. 15.3)
Crystal size (min, max) (mm)	0.05, 0.14	0.05, 0.14
Absorption correction	Analytical (*CrysAlisPro*; version 42.49)	
µ (mm^−1^)	16.34	16.34
2θ (°)	3.47–29.85	3.47–30.09
*h*, *k*, *l*	−13/12, −10/11, −20/20	−13/12, −10/11, −20/20
*m*	–	−1/1
No. of reflections	9583	29361
No. of independent reflections	1592	4780
No. of independent reflections [*I* > 3σ(*I*)]	1154	2603
*R*_int_ (all / *I* > 3σ)	0.0436 / 0.055	0.074 / 0.0824

Refinement	Full-matix least-squares on *F*^2^	
Weighting σ	0.03	
*R*_1_ (*I* > 3σ)	0.0371	0.0419 / 0.0286 / 0.0630†
*wR*_1_ (*I* > 3σ)	0.0690	0.0765 / 0.0542 / 0.1131†
*R*_2_ (all)	0.0611	0.1061 / 0.0517 / 0.1766†
*wR*_2_ (all)	0.0774	0.0984 / 0.0635 / 0.1514†
No. of parameters	100	226
No. of constraints	–	7
GoF (*I* > 3σ)	1.4180	1.2475
GoF (all)	1.3388	1.1627
Δρ_max_ (e Å^−3^)	1.95	1.22
Δρ_min_ (e Å^−3^)	−1.98	−1.18

Data collection and absorption correction: *CrysAlisPro* (Rigaku Oxford Diffraction, 2020 ▸); refinement: *Jana2020* (Version 2.1 29/8/2025) (Petříček *et al.*, 2023 ▸).

† Values are given for all / main / first-order satellite reflections.

**Table 2:** Interatomic distances *d* and bond valence sums (bvs) for the average three-dimensionally periodic crystal structure of Rb_2_[Si_2_O_5_]

	*d* (Å)	bvs (v.u.)
Si1_av_–O4_av_^i^	1.489 (6)	
Si1_av_–O1_av_	1.520 (4)	
Si1_av_–O3_av_	1.597 (4)	
Si1_av_–O6_av_	1.661 (7)	
Si1_av_–O7_av_	1.722 (6)	
Si1_av_–O4_av_	1.790 (6)	3.93 (2)

Si2_av_–O5_av_^ii^	1.495 (6)	
Si2_av_–O2_av_	1.527 (4)	
Si2_av_–O3_av_^iii^	1.583 (4)	
Si2_av_–O6_av_	1.687 (6)	
Si2_av_–O7_av_	1.666 (6)	
Si2_av_–O5_av_	1.785 (6)	3.97 (2)

Rb1_av_–O2_av_^iv^	2.736 (4)	
Rb1_av_–O2_av_	2.876 (4)	
Rb1_av_–O1_av_^v^	2.909 (4)	
Rb1_av_–O5_av_^vi^	3.056 (6)	
Rb1_av_–O7_av_^v^	3.088 (6)	
Rb1_av_–O4_av_^vii^	3.145 (6)†	0.666 (3)

Rb2_av_–O1_av_^viii^	2.781 (4)	
Rb2_av_–O7_av_	2.849 (6)	
Rb2_av_–O1_av_^ix^	2.915 (4)	
Rb2_av_–O2_av_^ii^	2.918 (4)	
Rb2_av_–O6_av_^v^	2.958 (7)	
Rb2_av_–O5_av_^ii^	3.076 (6)	
Rb2_av_–O3_av_	3.089 (6)	
Rb2_av_–O4_av_^ix^	3.141 (6)†	0.830 (3)

Symmetry codes: (i) 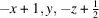; (ii) 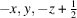; (iii) 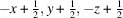; (iv) 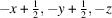; (v) 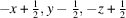; (vi) 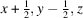; (vii) 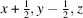; (viii) 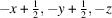; (ix) 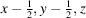.

† Next-nearest oxygen atom for Rb1_av_ and Rb2_av_ at 3.278 (8) and 3.569 (4), respectively.

**Table 3:** Interatomic distances *d*_Si–O_ (Å) including average (av) and extreme values caused by the modulation and bvs (v.u.) in the modulated, (3 + 1)-dimensional crystal structure of Rb_2_[Si_2_O_5_]

	*d*_av_	*d*_min_	*d*_max_
Si1*a*–O1	1.548 (14)	1.508 (17)	1.572 (17)
Si1*a*–O3	1.661 (15)	1.601 (17)	1.785 (17)
Si1*a*–O4*a*	1.634 (18)	1.57 (2)	1.67 (2)
Si1*a*–O5*a*	1.64 (2)	1.62 (3)	1.65 (3)
	bvs_av_	bvs_min_	bvs_max_
Si1*a*–O	4.07 (1)	3.95 (1)	4.17 (1)

Si1*b*–O1	1.559 (14)	1.528 (18)	1.598 (18)
Si1*b*–O3	1.632 (17)	1.54 (2)	1.699 (19)
Si1*b*–O4*a*^i^	1.65 (2)	1.62 (3)	1.69 (3)
Si1*b*–O5*b*	1.640 (19)	1.63 (3)	1.66 (3)
	bvs_av_	bvs_min_	bvs_max_
Si1*b*–O	4.08 (1)	3.87 (2)	4.33 (2)

Si2*a*–O2	1.560 (15)	1.533 (19)	1.606 (19)
Si2*a*–O3^ii^	1.634 (16)	1.601 (18)	1.723 (18)
Si2*a*–O4*b*	1.6424 (18)	1.60 (2)	1.69 (2)
Si2*a*–O5*b*	1.64 (2)	1.63 (3)	1.65 (3)
	bvs_av_	bvs_min_	bvs_max_
Si2*a*–O	4.08 (1)	3.86 (1)	4.16 (1)

Si2*b*–O2	1.541 (16)	1.49 (2)	1.59 (2)
Si2*b*–O3^ii^	1.640 (19)	1.59 (2)	1.70 (2)
Si2*b*–O4*b*^iii^	1.650 (19)	1.61 (3)	1.70 (3)
Si2*b*–O5*a*	1.62 (2)	1.61 (3)	1.63 (3)
	bvs_av_	bvs_min_	bvs_max_
Si2*b*–O	4.16 (1)	3.87 (2)	4.41 (2)

Symmetry codes: (i) 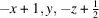; (ii) 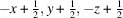; (iii) 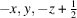.

**Table 4:** Interatomic distances *d*_Rb–O_ (Å) including average (ave) and extreme values caused by the modulation Bond valence sums (bvs) (v.u.) were calculated up a distance of 3.2 Å in the modulated, (3 + 1)-dimensional crystal structure of Rb_2_[Si_2_O_5_]

	*d*_av_	*d*_min_	*d*_max_	*t* range
Rb1–O1^i^	2.919 (3)	2.785 (6)	3.055 (6)	0.0–1.0
Rb1–O2	2.895 (6)	2.784 (6)	3.004 (6)	0.0–1.0
Rb1–O2^ii^	2.747 (6)	2.729 (7)	2.76 (1)	0.0–1.0
Rb1–O3	3.347 (16)	2.961 (17)	4.049 (17)	0.0–1.0
Rb1–O3	3.407 (14)	2.951 (15)	3.700 (15)	0.0–1.0
Rb1–O4*b*^iii^	3.020 (15)	2.979 (19)	3.151 (19)	0.0–0.31 and 0.82–1.0
Rb1–O4*a*	3.099 (13)	3.056 (17)	3.239 (17)	0.0–0.12 and 0.63–1.0
Rb1–O5*b*^i^	3.135 (15)	2.85 (2)	3.27 (2)	0.32–0.81
	bvs_av_	bvs_min_	bvs_max_	
Rb1–O	1.037 (1)	0.9625 (1)	1.1256 (1)	

Rb2–O1^iv^	2.902 (6)	2.699 (6)	3.111 (6)	0.0–1.0
Rb2–O1^v^	2.783 (6)	2.756 (7)	2.812 (7)	0.0–1.0
Rb2–O2^vi^	2.911 (6)	2.681 (6)	3.145 (6)	0.0–1.0
Rb2–O2^i^	3.582 (6)	3.147 (6)	4.008 (6)	0.0–1.0
Rb2–O3	3.118 (14)	3.048 (15)	3.171 (15)	0.0–1.0
Rb2–O5*b*	2.952 (16)	2.82 (2)	3.06 (2)	0.0–0.12 and 0.63–1.0
Rb2–O4*b*^vi^	3.029 (15)	2.966 (19)	3.108 (19)	0.13–0.62
Rb2–O5*a*^i^	3.046 (15)	2.92 (2)	3.21 (2)	0.0–0.31 and 0.82–1.0
Rb2–O4*a*^iv^	3.101 (14)	3.050 (18)	3.151 (18)	0.32–0.81
	bvs_av_	bvs_min_	bvs_max_	
Rb2–O	1.0601 (1)	0.9384 (2)	1.2390 (2)	

Symmetry codes: (i) 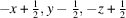; (ii) 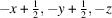; (iii) 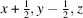; (iv) 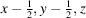; (v) 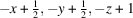; (vi) 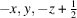.

**Table 5:** Related phyllosilicate phases with [4.8]^2^ nets Unit-cell parameters *a*, *b*, *c* rounded to three decimal places, β rounded to one decimal place.

Compound	Space group	*a* (Å)	*b* (Å)	*c* (Å)	β (°)	Mesh† (Å^2^)	Reference
Rb_2_[Si_2_O_5_]	*C*2/*c*	9.866 (1)	8.401 (1)	14.770 (1)	90.1 (1)	*ab*, 82.9	This study
Cs_2_[Si_2_O_5_]	*P*2_1_/*c*	10.061 (5)	8.609 (5)	8.414 (5)	122.76 (4)	*ab*, 86.6	de Jong *et al.* (1994 ▸)
KNa_2_Ca_2_[Si_8_O_19_(OH)]·6H_2_O	*P*2/*c*	13.704 (2)	6.576 (1)	13.751 (2)	105.8 (1)	*bc*, 90.4	Zubkova *et al.* (2009 ▸)
K_2_Ca[Si_4_O_9_(OH)]·3H_2_O	*P*2_1_/*c*	6.490 (1)	6.997 (1)	26.714 (2)	94.6 (1)	*ab*, 1/2 × 90.8	Zubkova *et al.* (2010 ▸)
K_2_Ca[Si_4_O_10_]·5H_2_O	*P*2_1_/*n*	6.493 (1)	6.992 (1)	32.087 (3)	94.7 (1)	*ab*, 1/2 × 90.8	Zubkova *et al.* (2010 ▸)

† Orientation and size of the corresponding mesh in the [SiO_4_] or [Si(O,OH)_4_] layer.

## Data Availability

Data supporting the results reported in your article can be accessed as published supporting material.
